# The Association between Depression and Perceived Stress among Parents of Autistic and Non-Autistic Children—The Role of Loneliness

**DOI:** 10.3390/ijerph19053019

**Published:** 2022-03-04

**Authors:** Kasper Sipowicz, Tadeusz Pietras, Marlena Podlecka, Łukasz Mokros

**Affiliations:** 1 Department of Interdisciplinary Disability Studies, The Maria Grzegorzewska University in Warsaw, Szczesliwicka 40, 02-353 Warsaw, Poland; ksipowicz@aps.edu.pl; 2 Second Department of Psychiatry, Institute of Psychiatry and Neurology in Warsaw, Sobieskiego 9, 02-957 Warsaw, Poland; tpietras@ipin.edu.pl; 3 Department of Neuroses, Personality Disorders and Eating Disorders, Institute of Psychiatry and Neurology in Warsaw, Sobieskiego 9, 02-957 Warsaw, Poland; mpodlecka@ipin.edu.pl; 4 Department of Clinical Pharmacology, Medical University of Lodz, Kopcinskiego 22, 90-153 Lodz, Poland

**Keywords:** depression, mood disorders, broad autism phenotype, social support

## Abstract

Having an autistic child significantly impairs the functioning of the family, including the wellbeing of the parents. The aim of this study was to assess whether loneliness mediates the relationship between perceived stress and the severity of depressive symptoms in the studied sample of parents. This cross-sectional study involved 39 parents of autistic children and 45 parents of non-autistic children. They completed a set of tests: a survey on sociodemographic and clinical data and psychometric questionnaires, i.e., Beck Depression Inventory II (BDI), De Jong Gierveld Loneliness Scale (DJGLS), and Perceived Stress Questionnaire (KPS). A rise in external and intrapsychic stress, independently, was linked to a rise in the severity of depressive symptoms. The severity of depression, loneliness and stress was higher among parents of autistic children compared with parents of non-autistic children. Intrapsychic stress exhibited an indirect effect through loneliness on the worsening of depressive symptoms.

## 1. Introduction

Autism is currently treated as a type of disability in which autistic people have problems with understanding and establishing social contacts. Traditional psychiatry treats the autism spectrum as a neurodevelopmental disorder [1]. Such an approach, although justified from the point of view of neurobiology, is strongly stigmatizing [2]. Therefore, autism is nowadays considered as a category of disability. Disability is a transactional category, to a large extent culturally conditioned, which develops at the intersection of biological phenomena and their social connotations. For example, autism may be considered as a manifestation of neurodiversity, not as a manifestation of pathology [1]. 

Families with autistic children can experience severe difficulties [3]. Autistic individuals often require care throughout their lives, and parents often fulfil this role [4]. Additionally, the family may often not receive support from the relatives and friends, who may not understand the behaviors of autistic children and move away from the family in deep crisis. There may appear also a breakdown of the existential narrative in the family. An autistic child often may not meet the parents’ expectations in terms of career development or starting his or her own family. This may be considered a major stressor for the nuclear family [5,6].

Loneliness is defined as a negative experience of perceived discrepancy between the existing and desired social relationships of an individual [7]. Loneliness appears to substantially increase the risk of depression, but the association appears to be bidirectional [8]. Experiencing loneliness may be perceived as a chronic stressor, and in turn chronic stress has been found to be risk factor for depression [9,10]. The caregivers of autistic children may experience high loneliness as a consequence of lack of social support [11]. People from the immediate vicinity of autistic people are known to be exposed to a higher incidence of affective disorders than that observed in the general population [12]. Additionally, loneliness was demonstrated to mediate the impact of stress on anxiety and depressive mood in a group of young healthcare workers exposed to the stress of the COVID-19 pandemic outbreak [13].

The aims of hereby research were to:

(1) Compare the sense of loneliness, depression and perceived stress between parents of autistic children and parents of non-autistic children.
(2) Assess whether perceived stress may have a meaningful indirect effect through loneliness on the severity of depressive symptoms in the studied sample of parents.

Based on the current literature, the following hypotheses were stated:

(1) Loneliness, depressive symptoms and perceived stress are likely to be higher among parents of autistic children than those of non-autistic children.
(2) Perceived stress is likely to have a meaningful indirect effect through loneliness on the severity of depressive symptoms in the studied sample of parents.

## 2. Materials and Methods

### 2.1. Study Group

This observational, cross-sectional study was conducted from 2019 to 2021 among mothers and fathers of ambulatory autistic patients of the Mental Health Outpatient Clinic in Aleksandrów Łódzki. If it was possible to examine both parents of a particular child, then only one person from the parental couple was selected randomly by drawing lots. The participants without an autistic child were gathered among adults who volunteered in response to an advertisement made by the researchers. Advertisements were placed to recruit parents of non-autistic children.

### 2.2. Inclusion and Exclusion Criteria

The inclusion criterion was informed consent for participation in the stud, having at least one autistic child (for the parents of autistic children) and having at least one child, none of which were autistic (for the parents of non-autistic children). The assumed age limit of the child was between 3 and 18 years old. The exclusion criteria comprised: lack of informed consent, serious and unstable somatic disease, a severe psychological trauma within six months preceding the study, and serious mental illness (e.g., schizophrenia or bipolar affective disorder or other psychotic disorder). 

### 2.3. Study Procedure

The study procedure involved completion of a set of self-reported questionnaires. The participants were assessed regarding the exclusion criteria by the researchers in the course of an interview. The diagnosis of autism was based on the DSM-5 criteria and was consistent with the ICD-10 classification applicable in Poland. The diagnosis of autism in the children of the parents forming the study group was established by a psychiatrist and employees of educational advisory and counselling centres functioning within the framework of the education system in Poland. The children from the non-autistic group were also verified regarding the diagnosis. After signing the informed consent, the participants were asked to complete a set of questionnaires: authors’ survey on sociodemographic and clinical data and psychometric questionnaires (described below). Upon completion of the questionnaires and clinical data, the patients were once again verified regarding the exclusion criteria. The study involved two groups: 39 mothers and fathers of autistic children and 45 parents of non-autistic children. The selected demographics of both groups are summarized in Table 1.

### 2.4. Operationalization of the Variables—Questionnaires

The Beck Depression Inventory version II (BDI) was utilized to assess the severity of depressive symptoms. The scale was adapted to Polish by Zawadzki et al. The test comprises 21 items considering the occurrence and intensity of depressive symptoms within past two weeks. Each item is scored from 0 to 3, which gives a total of 0 to 63 point. The higher the score, the greater the severity of depression [14,15]. 

Loneliness was assessed with the De Jong Gierveld Loneliness Scale (DJGLS) in a Polish adaptation by Grygiel et al. [16]. The questionnaire comprises eleven items. Each item consists of five-point answer scale. An increase in the DJGLS total score corresponds to a rise in the sense of loneliness [16].

The severity of stress was evaluated with the Perceived Stress Questionnaire (KPS) created by Plopa and Makarowski. The test consists of 27 statements, divided into four scales: emotional tension, intrapsychic stress, external stress and lie scale (or social desirability scale—a control scale to verify whether the respondents were truthful). Regarding the first three scales—an increase in the score indicates a rise in the severity of perceived stress [13].

The selected demographic details were collected in the form of a diagnostic survey—a questionnaire of the authors’ design. The questionnaire contained questions about age, sex, education level, place of residence, marital status, and number of children.

### 2.5. Statistical Analysis

The statistical analysis was conducted using Jasp ver 0.14.1 (University of Amsterdam, Amsterdam, The Netherlands). The categorical variables were presented as numbers with percentages. Pearson’s chi-squared test was used to assess the associations between variables in contingencies. The normality of the distribution of the variables was verified with the Shapiro–Wilk test and visual analysis of the histograms—none of the continuous variables met the assumption of normality of distribution in the studied sample. Thus, the continuous variables were characterized by their median value, with the first and third quartile, and the intergroup comparisons were assessed with Mann–Whitney’s U test. Benjamini–Hochberg correction, with an assumed false discovery rate of 0.25, was performed to avoid type 1 error due to multiple comparisons.

A mediation model was created, with the DJGLS score (loneliness) considered as a mediator. The severity of depressive symptoms, the BDI score, was considered as an outcome, whereas the KPS external stress, intrapsychic stress and emotional tension scores were considered as predictors. Each model was adjusted for the respondents’ age, sex, place of residence, having an autistic child and KPS Lie scale score. Pearson’s correlation quotient was used to verify the association between each two variables of interest. An a priori and sensitivity analysis was performed to verify the statistical power with G*Power 3.1 software. The power analysis was performed for a linear regression model, predicting the severity of depression, with 13 predictors in total (counting both independent variables of interest and variables that the model was controlled for), assuming a one-tailed test, level of significance at alpha = 0.05 and 95% power. The calculated minimal sample was 70. In sensitivity analysis, the estimated minimal meaningful effect was 0.13. Bootstrapping, with sampling set at *n* = 1000, was performed to empower the results and account for non-parametric distribution. The confidence intervals were computed using the bias-corrected percentile method as suggested by Biesanz et al. [14]. 

The size of effect was assessed with rank-biserial correlation quotient (for Mann–Whitney’s test) and with Cramer’s V quotient (in the case of a chi-squared test). Those coefficients may be interpreted in terms of Cohen’s thresholds for small (0.1), medium (0.3) and strong correlation (0.5). Statistical significance was defined as *p* < 0.05, or a confidence interval not encompassing 0.

## 3. Results

### 3.1. Intergroup Comparisons

The difference regarding parents’ sex, marital status, age, educational level and place of residence was statistically insignificant between the studied groups. Thus, it may be assumed that the groups were relatively matched regarding those variables (Table 1).

Parents of autistic children had higher scores in DJGLS compared with parents who do not have an autistic child (*p* = 0.038). This effect was of a strong size (R = 0.463). Additionally, there was a statistically significant difference in the KPS subscale score of intrapsychic stress (*p* = 0.019, with a strong effect size—R = 0.535), with parents who have a child with ASD scoring higher compared with parents without a child with ASD (Table 1). 

The difference regarding the BDI score, the KSP external stress score, the KPS emotional tension score and the KPS total score were not statistically significant, yet a tendency towards higher scores among parents of children with ASD than among parents who do not have a child with ASD can be noticed (Table 1). 

The differences regarding age, sex, marital status, education level, a total number of children were not statistically significant. See Table 1 for detailed results.

### 3.2. Mediation Analysis

**Figure 1:**
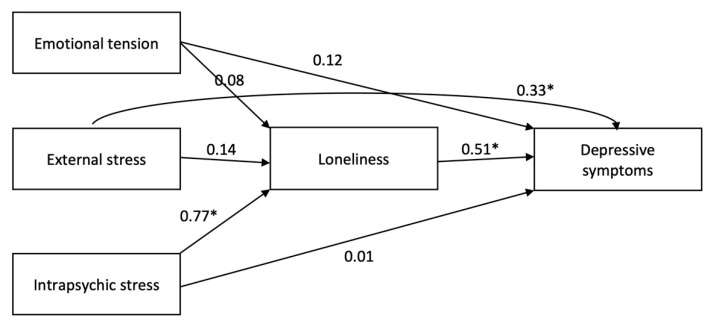
Simplified graphic illustration of the constructed model of the effect of perceived stress (measured with Perceived Stress Questionnaire, KPS) on the severity of depressive symptoms (i.e., Beck Depression Inventory score, BDI)—direct and indirect (mediated by loneliness, i.e., De Jong Giervald Loneliness Scale score, DJGLS) in the studied group of parents of children with and without autism spectrum disorder (ASD). The presented number indicate the standardized coefficients (size of effect). The model was adjusted for sex, age, place of residence and having a child with ASD and KPS lie score (i.e., social desirability). * statistically significant, based on 95% confidence interval.

The constructed mediation model had a high coefficient of determination (R^2^ = 0.871), which indicated that the constructed model explained 87% of variance of the BDI score. There was a statistically significant total effect of KPS external stress and intrapsychic stress on the BDI score—a rise in external and intrapsychic stress, independently, was linked to a rise in the severity of depressive symptoms. The effect of intrapsychic stress on BDI was exhibited through DJGLS score, whereas for KPS external stress the effect on BDI score was direct, not indirect through DJGLS score (see Table 2 and Figure 1). All the described effects were controlled for sex, age, having a child with ASD and place of residence. The correlations between the variables of interest can be seen in Table 3.

## 4. Discussion

Following from the analysis of data obtained in the study of our group, the severity of depression, loneliness and stress were higher among parents of autistic children than among parents of children without autism. We are aware that our group is not representative and was not recruited by random sampling. Nevertheless, our results are consistent with the literature data [12]. Scherer’s team conducted a meta-analysis of 19 English-language studies selected from among 5839 works. These research works were carried out on a sample characterized by high socio-economic status. About 70% of the studies involved parents of autistic children and children with cerebral palsy. Almost all the papers demonstrated an association between various types of disability in children and the occurrence of depression and anxiety in their parents. The severity of depression and anxiety in these parents correlated positively with the severity of disability in the children and with the low socio-economic status of these families. More than 30% of parents of children with intellectual disabilities meet the criteria for moderate depression, whereas that percentage among parents of healthy children amounts to 7% only. In turn, moderate anxiety occurs in 31% of parents of children with intellectual disabilities, whereas among parents of healthy children it is only 14%. The meta-analysis by Scherer et al. showed that having an autistic child or a child with cerebral palsy promotes moderately the occurrence of depression in the parents of such children [12]. 

Cohrs and Leslie showed that the relative risk of depression in mothers of autistic children is OR 2.95, 95% CI 2.81–3.09. For fathers, this coefficient is OR 2.41, 95% CI 2.25–2.58 [17]. 

A meta-analysis by Schnabel and others included 9208 parents of autistic children. As many as 33% of parents met the clinical criteria for depression, 10% for anxiety disorders, and 4% for obsessive compulsive disorder [18]. This research is one of the first attempts to study the psychopathology of parents of children with autism spectrum disorder on a large group. However, it lacks a comparison with the prevalence of psychiatric diagnoses in the group of parents of non-autistic children. It seems that the need for constant care of an autistic child, educational problems and social stigma are a strong and long-lasting stressor for parents, which can induce affective disorders and mood disorders in predisposed persons. 

Our current study shows that the level of loneliness in parents of autistic children is higher than that of parents of non-autistic children. It can be assumed that the presence of an autistic child at home, the need for constant care, stigmatization and, above all, the lack of understanding of people who do not have autistic children may intensify the social alienation of parents of autistic children, which is often manifested by the loneliness they feel [19,20,21]. Our observation, however, requires further in-depth studies in a larger group and taking into account other clinical variables. 

In addition, our research has shown that the effect of intrapsychic stress on an increase in depressive symptoms was indirect—through loneliness. The severity of external stress was associated with the severity of depression symptoms and this effect wad direct. Strong and prolonged stress induces a reduction in the severity of serotonergic and adrenergic transmission in the human and rodent brain, which are typical neurochemical changes in depression [22,23,24]. This should be explained by the fact that the stress response is not specific to the inducing stressor. Thus, external stress may induce depression regardless of whether having an autistic child was the stressor, or whether it was an external stressor of another type [25].

The current research has demonstrated that depression in parents was associated with an increase in external and intrapsychic stress, and that the effect of intrapsychic stress is expressed via loneliness. In view of the above, these results cannot be discussed only with respect to parents of autistic children. Additionally, parents of autistic children demonstrated statistically significantly higher severity of intrapsychic stress, but not that of external stress and emotional tension. This observation seems particularly interesting and surprising. One may be tempted to state that a child’s autism, the breakdown of the intra-family narrative and the change of life goals may be deeply internalized by parents of autistic children [26]. In addition, parents of autistic children may have the same genetic factors that favor communication disorders as their children. The severity of neuropsychological deficits in parents burdened genetically with autism is not significant enough to diagnose the ASD spectrum, but it may be a factor conducive to the development of depression and other mental disorders [27]. The described phenomenon was first detected in families of people with schizophrenia [28].

The shortcoming of our work is the small size of the studied sample, which, however, meets the requirements of the statistical minimum in the performed power analysis. Studies on a larger sample would allow to draw more unambiguous conclusions. We hope to continue the research in the future. Another limitation of our research is the lack of randomness of the studied samples. It does not take into account parents of autistic children with a low severity of disorders who do not seek the help of a specialist. Moreover, there was a statistically significant difference in the age of children between the groups, which may be due to non-randomness and no sample matching procedure introduced. Only cross-sectional epidemiological studies would enable us to develop a more objective model of the relationship between the studied variables. Additionally, only self-reported methods were employed and the use of such cannot be an equivalent of a clinical diagnosis, e.g., in terms of depression assessment. However, it should be noticed that the utilized questionnaires are widely recognized and utilized in clinical and research settings.

Moreover, the research results should be taken into account in the development of support programs for parents of autistic children. The therapeutic work should focus on reinforcement of the mechanisms of coping with intrapsychic stress. The birth of an autistic child changes the perception of the family narrative so much that it becomes an intrapsychic stressor, not an external one [29]. The above means that stress in parents of autistic children is due more to internalized social norms than to an objective situation. The interiorization of these norms and the negative image of a child with communication disorders in the parents’ eyes is their internal stressor resulting from their ideal image of the family, and not from an objective situation [29]. Equipping parents with the mechanisms of coping with such stress is, in the light of the obtained results, a key element of psychological support for that social group [30].

## 5. Conclusions

The severity of depression, loneliness and stress was higher among parents of autistic children than among parents of non-autistic children. Intrapsychic stress exhibited an indirected effect through loneliness on worsening of depressive symptoms. The effect of external stress on depression was direct among studied parents of autistic and non-autistic parents.

## Figures and Tables

**Table 1:** Comparison of the variables of interest between the parents having a child with autism spectrum disorder (ASD) and having children without ASD in the studied sample.

		Non-Autistic Child (*N* = 45)	Autistic Child (*N* = 39)	Statistical Tests
Age, Me (Q1–Q3)		34 (29–41)	39 (32–44)	W = 669.5, *p* = 0.134, R = 0.237
Sex	Women, *N* (%)	38 (84%)	32 (82%)	Chi^2^ = 0.086, df = 1, *p* = 0.250, V = 0.032
	Men, *N* (%)	7 (16%)	7 (18%)	
Place of residence	City	32 (71%)	29 (74%)	Chi^2^ = 0.111, df = 1, *p* = 0.231, V = 0.036
	Rural area	13 (29%)	10 (26%)	
Marital status, *N* (%)	Single	3 (7%)	5 (13%)	Chi^2^ = 6.789, df = 3, *p* = 0.115, V = 0.284
	Married	38 (84%)	25 (64%)	
	Divorced	3 (7%)	9 (23%)	
	Widowed	1 (2%)	0 (0%)	
Education level, *N* (%)	Secondary	20 (44%)	18 (46%)	Chi^2^ = 1.130, df = 2, *p* = 0.173, V = 0.116
	Vocational	3 (7%)	5 (13%)	
	Higher	22 (49%)	16 (41%)	
Number of children, *N* (%)	One	26 (58%)	25 (64%)	W = 910.0, *p* = 0.211, R = 0.037
	Two	16 (36%)	9 (23%)	
	Three	1 (2%)	4 (10%)	
	Four	2 (4%)	1 (3%)	
Age of autistic child, Me (Q1–Q3)		-	8 (5–10)	-
BDI score, Me (Q1–Q3)		7 (6–7)	7 (6–11)	W = 595.5, *p* = 0.096, R = 0.321
DJGLS score, Me (Q1–Q3)		12 (11–13)	15 (12–38)	W = 471.5, *p* = 0.038, R = 0.463
KPS scores, Me (Q1–Q3)	Emotional tension	21 (20–23)	23 (21–27)	W = 527.5, *p* = 0.076, R = 0.399
	External stress	23 (22–24)	23 (22–27)	W = 753.5, *p* = 0.154, R = 0.141
	Intrapsychic stress	22 (21–24)	25 (23–30)	W = 408.0, *p* = 0.019, R = 0.535
	Lie scale	18 (17–18)	18 (17–19)	W = 947.0, *p* = 0.192, R = 0.079
	total score	65 (63–70)	71 (67–83)	W = 521.0, *p* = 0.057, R = 0.406

Continuous variables are presented as median values (Me) with first and third quartile (Q1–Q3). Nominal variables are presented as number of observations (N) with percentage (%). BDI—Beck Depression inventory score, DJGLS—De Jong Gierveld Loneliness scale score, KPS—Perceived Stress Questionnaire; W—Mann–Whitney test W statistics, *p*—probability in the test after Benjamini-Hochberg correction, R—rank-biserial correlation (size of effect), Chi^2^—Chi-square test statistics, df—degrees of freedom, V—Cramer’s V statistics (size of effect).

**Table 2:** Summary of the assessment of the effect of perceived stress on the severity of depressive symptoms—direct and indirect (mediated by loneliness in the studied group of parents of children with and without autism).

Variable					Estimate	95% CI		z
**Direct Effects**								
Emotional tension		→		BDI score	0.122	−0.109	0.370	1.021
External stress		→		BDI score	0.326	0.047	0.564	3.637
Intrapsychic stress		→		BDI score	0.008	−0.227	0.297	0.056
**Indirect Effects**								
Emotional tension	→	DJGLS score	→	BDI score	0.043	−0.067	0.185	0.670
External stress	→	DJGLS score	→	BDI score	0.074	−0.027	0.203	1.487
Intrapsychic stress	→	DJGLS score	→	BDI score	0.393	0.203	0.694	3.864
**Total Effects**								
Emotional tension		→		BDI score	0.164	−0.098	0.435	1.221
External stress		→		BDI score	0.400	0.090	0.635	3.990
Intrapsychic stress		→		BDI score	0.401	0.119	0.736	3.034

Perceived stress—measured with Perceived Stress Questionnaire, KPS; the severity of depressive symptoms—measured with Beck Depression Inventory score, BDI; loneliness—measured with De Jong Giervald Loneliness Scale score, DJGLS. Results presented as standardized estimates (from linear regression models), with 95% Confidence Intervals (CI), derived from bootstrapping with *N* = 1000 sampling. All models were adjusted for age, sex, place of residence, having a child with ASD and KPS Lie scale score (social desirability). z—Sobel test statistics. →—direction of the tested path.

**Table 3:** Pearson correlation quotients between the continuous variables of interest in the group of parents of autistic and non-autistic children.

	1. BDI	2	3	4	5	6
2. Loneliness	0.890 **	—				
3. External stress	0.866 **	0.804 **	—			
4. Emotional tension	0.867 **	0.870 **	0.877 **	—		
5. Intrapsychic stress	0.860 **	0.900 **	0.828 **	0.914 **	—	
6. Social desirability	−0.235 *	−0.138	−0.185	−0.136	−0.089	—
7. Stress total score	0.902 **	0.898 **	0.932 **	0.974 **	0.960 **	−0.136

Perceived stress (external, intrapsychc and emotional tension)—measured with Perceived Stress Questionnaire, KPS; the severity of depressive symptoms—measured with Beck Depression Inventory score, BDI; loneliness—measured with De Jong Giervald Loneliness Scale score, DJGLS, * *p* < 0.05, ** *p* < 0.001.

## Data Availability

The data presented in this study are available on request from the corresponding author.

## References

[1] Zhao Y., Yang L., Gong G., Cao Q., Liu J. (2022). Identify Aberrant White Matter Microstructure in ASD, ADHD and Other Neurodevelopmental Disorders: A Meta-Analysis of Diffusion Tensor Imaging Studies. Prog. Neuro-Psychopharmacol. Biol. Psychiatry.

[2] Dwyer P. (2022). Stigma, Incommensurability, or Both? Pathology-First, Person-First, and Identity-First Language and the Challenges of Discourse in Divided Autism Communities. J. Dev. Behav. Pediatr. JDBP.

[3] Karst J.S., van Hecke A.V. (2012). Parent and Family Impact of Autism Spectrum Disorders: A Review and Proposed Model for Intervention Evaluation. Clin. Child Fam. Psychol. Rev..

[4] Thompson C., Bölte S., Falkmer T., Girdler S. (2018). To Be Understood: Transitioning to Adult Life for People with Autism Spectrum Disorder. PLoS ONE.

[5] Hoogsteen L., Woodgate R.L. (2013). Centering Autism Within the Family: A Qualitative Approach to Autism and the Family. J. Pediatr. Nurs..

[6] Heaphy B. (2018). Reflexive Convention: Civil Partnership, Marriage and Family. Br. J. Sociol..

[7] Cacioppo J.T., Hawkley L.C., Leary R., Hoyle R.H. (2009). Loneliness. Handbook of Individual Differences in Social Behavior.

[8] Cacioppo J.T., Hughes M.E., Waite L.J., Hawkley L.C., Thisted R.A. (2006). Loneliness as a Specific Risk Factor for Depressive Symptoms: Cross-Sectional and Longitudinal Analyses. Psychol. Aging.

[9] Moseley R.L., Turner-Cobb J.M., Spahr C.M., Shields G.S., Slavich G.M. (2021). Lifetime and Perceived Stress, Social Support, Loneliness, and Health in Autistic Adults. Health Psychol..

[10] Campagne D.M. (2019). Stress and Perceived Social Isolation (Loneliness). Arch. Gerontol. Geriatr..

[11] Lei X., Kantor J. (2021). Social Support and Family Functioning in Chinese Families of Children with Autism Spectrum Disorder. Int. J. Environ. Res. Public Health.

[12] Scherer N., Verhey I., Kuper H. (2019). Depression and Anxiety in Parents of Children with Intellectual and Developmental Disabilities: A Systematic Review and Meta-Analysis. PLoS ONE.

[13] Bonilla-Sierra P., Manrique-G A., Hidalgo-Andrade P., Ruisoto P. (2021). Psychological Inflexibility and Loneliness Mediate the Impact of Stress on Anxiety and Depression Symptoms in Healthcare Students and Early-Career Professionals During COVID-19. Front. Psychol..

[14] Beck A.T., Steer R.A., Brown G.K. (1996). BDI-II. Beck Depression Inventory.

[15] Zawadzki B., Popiel A., Pragłowska E. (2009). Charakterystyka Psychometryczna Polskiej Adaptacji Kwestionariusza Depresji BDI-II Aarona T. Becka. Psychol. Etol. Genet..

[16] Grygiel P., Humenny G., Rebisz S., Świtaj P., Sikorska J. (2013). Validating the Polish Adaptation of the 11-Item De Jong Gierveld Loneliness Scale. Eur. J. Psychol. Assess..

[17] Cohrs A.C., Leslie D.L. (2017). Depression in Parents of Children Diagnosed with Autism Spectrum Disorder: A Claims-Based Analysis. J. Autism Dev. Disord..

[18] Schnabel A., Youssef G.J., Hallford D.J., Hartley E.J., McGillivray J.A., Stewart M., Forbes D., Austin D.W. (2020). Psychopathology in Parents of Children with Autism Spectrum Disorder: A Systematic Review and Meta-Analysis of Prevalence. Autism.

[19] Recio P., Molero F., García-Ael C., Pérez-Garín D. (2020). Perceived Discrimination and Self-Esteem among Family Caregivers of Children with Autism Spectrum Disorders (ASD) and Children with Intellectual Disabilities (ID) in Spain: The Mediational Role of Affiliate Stigma and Social Support. Res. Dev. Disabil..

[20] Salleh N.S., Abdullah K.L., Yoong T.L., Jayanath S., Husain M. (2020). Parents’ Experiences of Affiliate Stigma When Caring for a Child with Autism Spectrum Disorder (ASD): A Meta-Synthesis of Qualitative Studies. J. Pediatr. Nurs..

[21] Zhou T., Wang Y., Yi C. (2018). Affiliate Stigma and Depression in Caregivers of Children with Autism Spectrum Disorders in China: Effects of Self-Esteem, Shame and Family Functioning. Psychiatry Res..

[22] Ménard C., Hodes G.E., Russo S.J. (2016). Pathogenesis of Depression: Insights from Human and Rodent Studies. Neuroscience.

[23] Dean J., Keshavan M. (2017). The Neurobiology of Depression: An Integrated View. Asian J. Psychiatry.

[24] Park C., Rosenblat J.D., Brietzke E., Pan Z., Lee Y., Cao B., Zuckerman H., Kalantarova A., McIntyre R.S. (2019). Stress, Epigenetics and Depression: A Systematic Review. Neurosci. Biobehav. Rev..

[25] Pizzagalli D.A. (2016). Psychobiology of the Intersection and Divergence of Depression and Anxiety. Depress. Anxiety.

[26] Kandel I., Merrick J. (2003). The Birth of a Child with Disability. Coping by Parents and Siblings. Sci. World J..

[27] Kose S., Bora E., Erermiş S., Özbaran B., Bildik T., Aydın C. (2013). Broader Autistic Phenotype in Parents of Children with Autism: Autism Spectrum Quotient–Turkish Version. Psychiatry Clin. Neurosci..

[28] Nicodemus K.K., Callicott J.H., Higier R.G., Luna A., Nixon D.C., Lipska B.K., Vakkalanka R., Giegling I., Rujescu D., St. Clair D. (2010). Evidence of Statistical Epistasis between DISC1, CIT and NDEL1 Impacting Risk for Schizophrenia: Biological Validation with Functional Neuroimaging. Hum. Genet..

[29] Hsiao Y.-J., Higgins K., Pierce T., Whitby P.J.S., Tandy R.D. (2017). Parental Stress, Family Quality of Life, and Family-Teacher Partnerships: Families of Children with Autism Spectrum Disorder. Res. Dev. Disabil..

[30] Spain D., Sin J., Paliokosta E., Furuta M., Prunty J.E., Chalder T., Murphy D.G., Happé F.G. (2017). Family Therapy for Autism Spectrum Disorders. Cochrane Database Syst. Rev..

